# Regional Variation in Comorbid Prediabetes and Diabetes and Associated Factors among Hypertensive Individuals in Rural Bangladesh, Pakistan, and Sri Lanka

**DOI:** 10.1155/2019/4914158

**Published:** 2019-04-30

**Authors:** Liang Feng, Aliya Naheed, H. Asita de Silva, Imtiaz Jehan, Rubhana Raqib, Md Tauhidul Islam, Nathasha Luke, Anuradhani Kasturiratne, Hamida Farazdaq, Sahar Senan, Tazeen H. Jafar, COBRA-BPS Study Group

**Affiliations:** ^1^Program in Health Services and Systems Research, Duke-NUS Medical School, Singapore; ^2^Initiative for Non-Communicable Diseases, Health Systems and Population Studies Division, icddr, b, Dhaka, Bangladesh; ^3^Clinical Trials Unit, Department of Pharmacology, Faculty of Medicine, University of Kelaniya, Kelaniya, Sri Lanka; ^4^Department of Community Health Science, Aga Khan University, Karachi, Pakistan; ^5^Immunobiology, Nutrition and Toxicology Laboratory, Infectious Diseases Division, icddr, b, Dhaka, Bangladesh; ^6^Department of Public Health, Faculty of Medicine, University of Kelaniya, Kelaniya, Sri Lanka; ^7^Department of Family Medicine, Aga Khan University, Karachi, Pakistan; ^8^Department of Renal Medicine, Singapore General Hospital, Singapore; ^9^Duke Global Health Institute, Duke University, Durham, NC, USA

## Abstract

We aimed to explore the cross-country variation in the prevalence of comorbid prediabetes or diabetes and determine the sociodemographic, lifestyle, and clinical factors, especially body mass index (BMI) and waist circumference, associated with comorbid diabetes in individuals with hypertension in rural South Asia. We analyzed cross-sectional data of 2426 hypertensive individuals of ≥40 years from 30 randomly selected rural communities in Bangladesh, Pakistan, and Sri Lanka. Prediabetes was defined as fasting plasma glucose (FPG) between 100 and 125 mg/dL without use of antidiabetic treatment and diabetes as FPG ≥126 mg/dL or use of antidiabetic medication. The prevalence (95% CI) of prediabetes or diabetes (53.5% (51.5%, 55.5%)) and diabetes (27.7% (25.9%, 29.5%)) was high in the overall hypertensive study population in rural communities in 3 countries. Rural communities in Sri Lanka had the highest crude prevalence of prediabetes or diabetes and diabetes (73.1% and 39.3%) with hypertension, followed by those in Bangladesh (47.4% and 23.1%) and Pakistan (39.2% and 20.5%). The factors independently associated with comorbid diabetes and hypertension were residing in rural communities in Sri Lanka, higher education, international wealth index, waist circumference, pulse pressure, triglyceride, and lower high-density lipoprotein. The association of diabetes with waist circumference was stronger than with BMI in hypertensive individuals. Prediabetes or diabetes are alarmingly common among adults with hypertension and vary among countries in rural South Asia. The high prevalence of comorbid diabetes in Sri Lanka among hypertensives is not fully explained by conventional risk factors and needs further etiological research. Urgent public health efforts are needed to integrate diabetes control within hypertension management programs in rural South Asia, including screening waist circumference.

## 1. Introduction

Diabetes is a major global public health concern and a common comorbidity in individuals with hypertension [1, 2]. About 425 million people had diabetes worldwide in 2017, and this number is expected to increase to 629 million by 2045 [3]. The excess costs due to diabetes on healthcare systems alone are tremendous, primarily due to the grave consequences of microvascular and macrovascular complications (e.g., blindness, cardiovascular disease, myocardial infarction, stroke, and kidney failure) [2, 3]. The International Diabetes Federation estimated the global health expenditure on diabetes was 850 billion in 2017 [3]. Of all the individuals with diabetes worldwide, approximately 80% live in low- and middle-income countries [3].

South Asian countries have an increasing burden of diabetes [1, 4], with the estimated number of diabetic patients increasing from 58.7 million in 2010 to 101 million in 2030 [1]. The International Diabetes Foundation estimated diabetes-related healthcare expenditure in 2017 to be approximately US $9.5 billion in the South Asian region [3]. The rising burden could be related to increased life expectancy, rapid population growth, unplanned urbanization, and limited healthcare expenditure [5].

Studies in the West have consistently shown that type 2 diabetes is more common in South Asian immigrants than other ethnic groups [6–9]. Moreover, prediabetes tends to progress faster to diabetes in South Asians, at an earlier age than in Europeans [10]. Diabetes is also associated with greater risk of retinal and cerebral microvascular disease in South Asians than Europeans [11, 12]. The susceptibility of South Asians to dysglycemia has been shown to be apparent since early childhood and is associated with low birth weight and adverse in utero environment due to poor maternal nutrition, followed by excessive relative weight gain during childhood that persists into adulthood [13].

A high prevalence of diabetes has been reported in urban South Asia and has been associated with sedentary lifestyle and greater consumption of fast food rich in sugar and saturated fats associated with progressive social, cultural, and economic globalization [14, 15]. However, health systems are weaker, and complications from diabetes have worse outcomes in rural than in urban areas [16].

Diabetes frequently coexists with hypertension and reportedly affects 7.5% to 32% hypertensive individuals [17–23]. Studies of people with hypertension [19, 20, 23–26] identified similar risk factors for diabetes to those in general population [27, 28] such as demographical factors (older age and male gender), unhealthy life style (smoking and physical inactive), and clinical factors (overweight or central obesity and dyslipidemia). However, there is a dearth of studies comparing cross-country prevalence and determinants of diabetes among hypertensive individuals living in rural South Asia.

We analyzed baseline data from the ongoing COBRA-BPS (Control of Blood Pressure (BP) and Risk Attenuation-Bangladesh, Pakistan, and Sri Lanka) trial on 2426 hypertensive individuals, to examine the prevalence and cross-country differences in comorbid prediabetes and/or diabetes in hypertensive individuals in rural communities in 3 South Asian countries [29]. We aimed to determine whether the sociodemographic characteristics, lifestyle factors, obesity, and other clinical risk factors accounted for potential differences in prevalence of comorbid diabetes among individuals with hypertension in rural areas across the 3 South Asian countries. We also sought to determine whether central or generalized obesity was a stronger determinant of comorbid diabetes and hypertension. As an ancillary aim, we compared the awareness and management of diabetes in the 3 countries.

We hypothesized that (1) the prevalence of comorbid prediabetes or diabetes is high and varies among hypertensive individuals in rural communities across the three South Asian countries; (2) the cross-country variation in comorbid diabetes and hypertension will only partially be accounted for by differences in sociodemographic, lifestyle, and cardiovascular risk factors; and (3) waist circumference will be more strongly associated with comorbid diabetes and hypertension compared to body mass index (BMI).

## 2. Methods

### 2.1. Population

The present study was performed using baseline data from COBRA-BPS full-scale study. The detailed information on the study has been described earlier [29]. Briefly, COBRA-BPS full-scale study is an ongoing two-year cluster randomized controlled trial among 2643 hypertensive adults from 30 randomly selected rural clusters (communities), 10 clusters each, in Bangladesh, Pakistan, and Sri Lanka. In each country, cluster selection was stratified by distance (≤2.5 km for near and >2.5 for far) from the government primary care clinics, such that there were 6 near and 4 far clusters in each country. Individuals in each cluster were screened using door-to-door sampling method. The inclusion criteria for COBRA-BPS were age ≥40 years, hypertension (defined as sustained elevation of systolic blood pressure (SBP) to ≥140 mmHg, or diastolic blood pressure (DBP) to ≥90 mmHg based on two readings from 2 separate days, or receiving antihypertensive medications), and residents in the selected clusters. Individuals were excluded if they had severe physical incapacity, were pregnant, had advanced diseases (on dialysis, liver failure, and other systemic diseases), or were mentally comprised leading to incapability of giving consent.

Supplementary Figure S1 shows the study flow diagram. Of the 2977 hypertensive individuals from 30 randomly selected clusters in 3 countries, 2643 were enrolled in the clinical trial after excluding 334 individuals for various reasons (Supplementary Figure S1). Of the 2643 hypertensives recruited, 217 (9.3%) were excluded because they missed laboratory data on fasting blood glucose and were not on antidiabetic medication. The study protocol was approved by respective Ethical Review Committee in Singapore, Bangladesh, Pakistan, Sri Lanka, and UK. All study participants provided written informed consent.

### 2.2. Measurements

Self-reported sociodemographic and economic status (age, gender, education, marital status, international wealth index (IWI) [30]), lifestyle characteristics (smoking and physical activity), cardiovascular disease (CVD) (self-reported heart disease or stroke), and current medication use were obtained at the baseline visit. Physical activity was evaluated by the short version of the International Physical Activity Questionnaire (IPAQ) [31]. Framingham CVD risk score was computed, and ≥20% indicated a high global CVD risk at 10 years [32].

An overnight fasting blood sample was collected to measure lipids, serum creatinine, and plasma glucose (measured on Roche Hitachi-Cobas c311 in Bangladesh, Siemens ADVIA 1800 in Pakistan, and Beckman Coulter in Sri Lanka). Urine albumin and creatinine excretion were measured on spot urine samples by nephelometry using the array systems method on the same equipment as for blood tests in each country. All tests were done in an accredited laboratory in each country. Glomerular filtration rate (GFR) was estimated using the original Chronic Kidney Disease Epidemiology Collaboration (CKD-EPI) equation [33]. Urine albumin and creatinine ratio (UACR) was determined by urine albumin divided by urine creatinine. Chronic kidney disease was defined as the presence of estimated glomerular filtration rate (eGFR) ≤60 ml/min/1.73 m^2^ or UACR ≥30 mg/g.

On enrolment, participants' weight, height, waist circumference, and BP were measured. BMI was calculated as weight (in kilogram)/height (in meters^2^). BP was measured four times every 5 minutes of rest in sitting position using an Omron HEM-7300 digital monitor. The mean of last 2 readings was used in the analysis. Pulse pressure (PP) was calculated as the difference between SBP and DBP.

### 2.3. Analysis Methods

The main outcome was diabetes defined as a fasting plasma glucose (FPG) ≥126 mg/dL or self-reported use of antidiabetic medication. Prediabetes was defined as an FPG between 100 and 125 mg/dL and not on antidiabetic treatment.

Comparison of characteristics between hypertensive individuals with and without comorbid diabetes was performed using *t*-test for continuous variables and chi-square test for categorical variables. When continuous variables were not normally distributed, the Mann–Whitney *U* test was used. Age- and gender-standardized prevalence of comorbid prediabetes or diabetes with hypertension and only comorbid diabetes with hypertension was computed using direct standardization, with the standards being the age (grouped as 40∼50, 50∼60, 60∼70, and ≥70 years) and gender distribution of the total population. In addition, we examined the distribution of central obesity (waist circumference: ≥90 cm for male and ≥80 cm for female) [34] and categorical BMI (grouped as underweight or normal (<23.0 kg/m^2^) vs. overweight or generalized obesity (≥23.0 kg/m^2^)) [35] for hypertensive individuals with prediabetes or diabetes, and only diabetes.

We performed logistic regression analysis to evaluate the association between risk factors and comorbid diabetes in the entire sample of hypertensive individuals from 3 countries, allowing for within-cluster correlation. Covariates were chosen for the analysis if they were reported to be risk factors for diabetes in previous studies or were associated with diabetes based on standard logistic regression ignoring clustering effects. Covariates in the initial standard logistic regression model were country, socioeconomic variables (age, gender, education, marital status, and IWI), smoking, physical activity, BMI, waist circumference, PP, high-density lipoprotein (HDL), and triglycerides. We used stepwise method to select covariates with a significant level of 0.15 for retaining variables and of 0.10 for removing a variable. Country, education level, IWI, waist circumference, PP, HDL, and triglyceride were selected. We also retained all the excluded variables for final analysis because they were found to be risk factors for diabetes in previous studies [20, 25, 27, 28, 36].

Four models were constructed with these covariates. In model 1, only country was included; in model 2, we further introduced socioeconomic variables (age, gender, education, marital status, and IWI) and BMI (grouped as <18.5, 18.5∼23.0, 23.0∼27.5, and ≥27.5 kg/m^2^) [35]; model 3 further included waist circumference (grouped via gender-specific quartiles: for male ≤82, 82∼91, 91∼98, and ≥98 cm and for female ≤79, 79∼88, 88∼95, and ≥95 cm); and the final model was adjusted for the variables in model 3 plus smoking, physical activity (grouped via tertiles: <1381, 1381∼5544, and ≥5544 MET-min/week), PP, HDL, and triglycerides. All the models included cluster-specific random intercepts to account for within-cluster correlation.

We also investigated two-way interactions of country and gender with other variables to assess the presence of country- or gender-specific effect, respectively. Significant interactions were interpreted by the ratio of odds ratios (ROR) [37] and subgroup analysis. Finally, we computed the proportion (95% CI) of diabetic individuals with hypertension who (1) were aware of their diabetes status defined as self-reported doctor diagnosis of diabetes, (2) controlled BP to conventional target of <140/90 mmHg, (3) were receiving statin therapy, and (4) were receiving antidiabetic medications (number and different classes). All analyses were conducted using SAS version 9.4, and all hypothesis testing was 2-tailed with *P* < 0.05 set as statistically significant.

## 3. Results

### 3.1. Characteristics of Participants

Of all the 2643 individuals with hypertension, 2426 (91.8%) were included in the study. The mean age (±SD) of the participants was 58.8 (±11.3) years, the mean (±SD) BMI was 24.8 (±5.0) kg/m^2^, and the mean (±SD) waist circumference was 90.5 (±12.7) cm for male and 87.1 (±12.9) for female (Table 1).

Compared with hypertensive individuals without comorbid diabetes, those with comorbid diabetes were older, had a higher education and wealth index score, lower level of physical activity, and higher BMI, waist circumference, and PP, and were more likely to be from Sri Lanka. Comorbid diabetes was also positively associated with higher levels of triglyceride, CVD, CKD, and a CVD risk of 20% or above (Table 1). No significant association was found between comorbid diabetes and other variables.

Compared with individuals included in the analysis (*n*=2426), those excluded (*n*=217) were more likely from Pakistan, had lower education and lower proportion of married persons, and were at lower socioeconomic level and more often physically inactive (Supplementary Table S1).

### 3.2. Prevalence of Comorbid Prediabetes or Diabetes and Comorbid Diabetes with Hypertension Stratified by Country

Table 2 summarizes the crude, and age- and gender-standardized prevalence (95% CI) of comorbid prediabetes or diabetes, and comorbid diabetes in the overall rural hypertensive sample and by each country. The crude prevalence (95% CI) of comorbid prediabetes or diabetes among hypertensive individuals was 73.1% (70.0%, 76.2%) in Sri Lanka, 47.4% (44.0%, 50.7%) in Bangladesh, and 39.2% (35.6%, 42.8%) in Pakistan.

Likewise, comorbid diabetes was the most prevalent among hypertensives in Sri Lanka (39.3% (35.9%, 42.7%)), followed by Bangladesh (23.1% (20.2%, 25.9%)), and then Pakistan (20.5% (17.6%, 23.5%)), with almost no change in prevalence after adjustment for confounding by age and gender.

Supplementary Tables S2 and S3 report baseline characteristics by country and by comorbid diabetes status in each country, respectively.

### 3.3. BMI, Central Obesity, Comorbid Prediabetes or Diabetes and Hypertension

Of all the hypertensive individuals with comorbid prediabetes or diabetes, 71.5% (*n*=915) were overweight or had generalized obesity; 75.8% (*n*=981) were centrally obese; and 65.1% (*n*=833) were both overweight/generalized obesity plus central obesity. Of all the hypertensive individuals with comorbid diabetes, 74.9% (*n*=492) were overweight or had generalized obesity; 82.4% (*n*=552) were centrally obese; and 70.3% (*n*=462) were both overweight/generalized obesity plus central obesity (data not shown in the table).

### 3.4. Risk Factors for Comorbid Diabetes and Hypertension

The factors associated with comorbid diabetes in multivariable models are shown in Table 3. In model 1, compared with hypertensive individuals from rural areas in Pakistan, those from rural Bangladesh had similar odds of comorbid diabetes (OR = 1.16, 95% CI (0.84, 1.61)), while those from rural Sri Lanka had significantly greater odds of comorbid diabetes (OR = 2.55, 95% CI (1.86, 3.49)). Adjustment for sociodemographic and clinical factors in models 2, 3, and 4 did not alter the association.

BMI had a strong positive association with comorbid diabetes in model 2, but this association became nonsignificant with adjustment for waist circumference and other covariates in models 3 and 4.

Significant interactions were detected between country and marital status (*P* for interaction = 0.011), international wealth index (*P* for interaction = 0.038), and HDL (*P* interaction = 0.002), but no interaction with gender was significant.  Table 4 presents the results of multivariate analysis stratified by countries. There was a protective effect of being unmarried (vs. married) on comorbid diabetes only among hypertensives in rural Pakistan (OR = 0.44, 95% CI (0.24, 0.82)) (Table 4). IWI had a stronger positive association with comorbid diabetes in Pakistan (ROR = 1.47, 95% (1.07, 2.01)) and Bangladesh (ROR = 1.36, 95% (0.99, 1.86)) than Sri Lanka (Supplementary Table S4), but no association in Sri Lanka (Table 4). The inverse association between HDL and comorbid diabetes was only observed in Sri Lanka (OR = 0.80, 95% CI (0.73, 0.88)) (Table 4).

### 3.5. Awareness and Management of Comorbid Diabetes and Hypertension

Of all the 673 individuals with comorbid diabetes in hypertension, 74.3% knew that they had diabetes. The rates of awareness of comorbid diabetes were comparable between Bangladesh (81.6%) and Sri Lanka (80.0%), being much higher than that of Pakistan (52.6%). Only 32.8% controlled their BP under 140/90 mmHg, and 23.8% were on statin (Supplementary Table S5). About 69% of all hypertensive individuals with comorbid diabetes were on glucose-lowering medication, with the lowest prevalence in Pakistan (46%) (Supplementary Table S6). Overall, 48.7% of the hypertensive individuals with comorbid diabetes reported using biguanides, with 35.3% in Bangladesh, 32.2% in Pakistan, and 65% in Sri Lanka.

## 4. Discussion

Our analysis of data on 2426 hypertensive individuals from 30 randomly selected rural communities in Bangladesh, Pakistan, and Sri Lanka revealed a strikingly high prevalence of comorbid prediabetes or diabetes affecting 1 in 2 in hypertensive adults in 3 countries, and this was much higher in Sri Lanka (2 in 3 adults) than the other 2 countries. Similar cross-country variation was observed in the prevalence of comorbid diabetes with 1 in 4 hypertensive adults affected in all 3 countries and at least 1 in 3 in Sri Lanka. Formal education, higher IWI, higher waist circumference, elevated PP, increased levels of triglyceride, and residing in rural Sri Lanka (vs. in rural Pakistan), each, were significantly associated with higher odds of comorbid diabetes. Waist circumference was a stronger determinant of comorbid diabetes and hypertension than BMI. Higher HDL, by contrast, was associated with lower odds of comorbid diabetes and hypertension. These factors only partially explained the higher prevalence of comorbid diabetes among hypertensive individuals in rural Sri Lanka. Our findings of an alarmingly high prevalence of prediabetes and/or diabetes as a key comorbidity in individuals with hypertension call for integrating diabetes care with hypertension management program in rural areas in South Asia. Such efforts must be complemented with population-wide policy initiatives for reducing key risk factors especially obesity.

To our knowledge, this is the first report of cross-country comparison of prevalence of comorbid diabetes among rural community dwellers with hypertension in South Asian countries using a common protocol. We found that adults with hypertension in rural Sri Lanka were older, better educated, more centrally obese and less physically active, had higher socioeconomic status, and higher pulse pressure compared to those in rural Pakistan or Bangladesh (Supplementary Table S2). However, these risk factors could not account for the higher prevalence of comorbid diabetes among hypertensives in rural Sri Lanka compared to Pakistan or Bangladesh. An earlier study of urban hypertensive Pakistanis has shown that 27.6% had comorbid diabetes, which is higher than our finding of rural hypertensive Pakistanis (20.5%) and parallels that of all the three countries combined (28%) [18]. Ethnic differences in diabetes prevalence have previously been suggested among South Asian countries [9, 38–42]. Earlier studies of nationally representative samples of the general population have found the prevalence of diabetes was 10.3% among Sri Lankans [38], 9.7% among Bangladeshis [39], and 5.4% among Pakistanis [40], respectively. Using cross-sectional data of 1,122,771 immigrants aged ≥20 years from South Asia living in Canada, a higher age- and gender-standardized prevalence of diabetes has been reported in the diaspora from Sri Lanka than those from Pakistan and India [41]. In another study of 16,288 individuals aged 20 and above, higher prevalence of diabetes and prediabetes was reported in Chennai (22.8% and 37.9%, respectively) and Delhi (25.2% and 47.6%) than in Karachi (16.3% and 31.1%) [42]. A more recent study of 431,765 migrant South Asians in Canada reported that the diabetes prevalence was the highest in Sri Lankan immigrants (26.8%), followed by those from Bangladesh (22.2%) and Pakistan (19.6%) [9]. It is likely that some other unmeasured risk factors could explain the higher prevalence of comorbid diabetes among hypertensives in Sri Lanka. Decades of conflict from the civil war ending in 2009 has been shown to have an emotional and psychological impact with high rates of anxiety and depression in individuals in Sri Lanka [43]. Epidemiological studies have implicated psychological stresses such as depression and early-life adversity as risk factors for diabetes [44]. Psychological stress may affect the development of diabetes through the release of catecholamines and glucocorticoids such as cortisol, resulting in increased hepatic glucose output, decreased insulin secretion and sensitivity, central accumulation of body fat, and inflammation, as well as through its adverse effects on behaviors including diet, physical activity, and adherence to medication [44, 45]. The observed difference could also result from differential distribution of other unmeasured factors such as inflammatory markers [46], unhealthy diet [28], family history of diabetes [27], organochlorine pesticide [47], or their interaction in this ethnic group. More etiological research is needed in Sri Lanka to understand the higher risk of comorbid diabetes in the population with hypertension. Nevertheless, our findings of an alarmingly high prevalence of comorbid diabetes (at least 1 in 4) in individuals with hypertension in communities in rural South Asia underscore the need for screening all adults with hypertension for diabetes and management of the latter in the primary care settings.

It is worth noting that central obesity (waist circumference) was a stronger determinant of comorbid diabetes than overweight/generalized obesity (BMI) in the overall population from 3 countries. Previous studies of hypertensive Chinese have shown that both BMI and waist circumference were correlates of diabetes with comparable strength of association [19, 23], but others reported similar results to ours [48, 49]. Notwithstanding the cross-sectional study design, our findings suggest the independent role of central obesity in diabetes development. BMI reflects total body mass, and waist circumference reflects abdominal obesity, largely a reflection of visceral fat. Abdominal adipose tissue has been shown to be metabolically active especially when oxygenation patterns are dysfunctional leading to pathogenesis of insulin resistance, and glucose intolerance, which is associated with adverse cardiovascular risk profile [50–52]. For a given BMI, South Asians have higher amounts of abdominal adipose and are more insulin resistant than Caucasians [53, 54]. Our findings underscore the importance of including waist circumference as a risk marker for diabetes perhaps even preferentially than BMI in community screening programs in South Asian populations.

Our findings of the association between high triglycerides and comorbid diabetes are also consistent with evidence in other populations [26]. Recent post hoc analysis of the Diabetes Prevention Program suggested benefit of lowering triglyceride on reducing new-onset diabetes [55]. It is interesting to note that comorbid diabetes was more prevalent in individuals in the higher IWI strata. However, higher prevalence of diabetes in higher socioeconomic status has also been reported in previous studies in the region [56]. At the same time, it is important to highlight that even the higher socioeconomic strata households in these rural communities have low international purchasing power parity [57]. Thus, financial access to quality treatment is limited, and adverse outcomes of diabetes have been shown to be prevalent across all socioeconomic strata in South Asia.

BP control and lipid-lowering are key for preventing diabetes-related vascular complications [58, 59]. We found that the vast majority of hypertensive adults with diabetes had poor BP control (67%) using the conventional target of <140/90 mmHg. The American Diabetes Association (ADA) 2016 Standards of Medical Care in Diabetes Standards and the National Institute for Health and Care Excellence (NICE) guidelines recommend biguanides as first-line glucose-lowering drug for all individuals with type 2 diabetes needing drug therapy [60, 61], and statins for all patients aged ≥40 years with diabetes [61, 62]. However, we observed that biguanides and statins were underprescribed in all three countries, especially in Bangladesh and Pakistan. Our findings underscore the urgent need for comprehensive diabetes management program for all rural communities in Sri Lanka, Bangladesh, Pakistan, and other South Asian countries.

The major strengths of our study are that we used a uniform study design, door-to-door sampling of individuals, random selection of clusters, consistent definition of variables and outcomes, and standardized study procedures in all 3 countries. There are several limitations. First, our study is a cross-sectional study, precluding any cause-effect relationship inference between risk factors and comorbid diabetes. However, cross-sectional design is appropriate for determining cross-country variation in prevalence of comorbid diabetes and potential factors associated with the variation which was our main objective. Second, we defined diabetes using FBG only and did not complement with oral glucose test or HbA1c test, possibly underestimating diabetes prevalence in our sample. However, venous FBG is considered acceptable for diagnosis of diabetes in epidemiological studies [39]. Third, information on some important factors such as family history of diabetes, psychological stress, and exposure to pesticide was not collected in the study. These and other unmeasured factors need to be evaluated in future studies. Fourth, chemistry analyzers and reagents used for our laboratory tests were different between the laboratories from each country. However, all laboratories were accredited to international standards (CAP accreditation for the laboratories in Bangladesh and Pakistan, and Bio-Rad for Sri Lanka), minimizing the cross-lab variation in these tests. Finally, our sample consisted of hypertensive participants aged ≥40 years sampled in a certain geographic location in each country. Regional differences in the prevalence of diabetes have been observed within a country in South Asia [4, 63, 64]. Therefore, the findings may not be generalizable to all the rural population, especially those free of hypertension or younger than 40 years in each country. However, the 30 clusters were randomly selected and were at least 7 km apart and stratified by distance from government clinic in each country ensuring a fair representation of varying access to healthcare and minimizing selection bias. Thus, we believe our findings are robust and generalizable to the regions where our study was conducted.

In conclusion, the prevalence of comorbid diabetes and prediabetes was alarmingly high among individuals with hypertension in rural communities in Bangladesh, Pakistan, and Sri Lanka. Formal education, higher IWI, higher waist circumference, elevated PP, increased levels of triglyceride, and lower HDL, each, were significantly associated with higher odds of comorbid diabetes. In addition, the higher prevalence of comorbid diabetes among hypertensives in rural Sri Lanka (compared to Pakistan) could not be explained by socioeconomic factors, lifestyle behaviors, or cardiovascular risk factors. Waist circumference was a stronger risk factor for comorbid diabetes than BMI. Moreover, lack of awareness of comorbid diabetes, poor BP control, and underprescription of statins and biguanides were common in all three countries. Further research is necessary to explore reasons for variation in the prevalence of comorbid diabetes across the countries, especially the high prevalence in rural Sri Lanka. Concerted efforts are needed to develop and implement effective and customized public health programs for prevention and management of comorbid diabetes integrated with hypertension care in rural South Asia.

## Figures and Tables

**Table 1:** Comparison of baseline characteristics between hypertensive individuals with and without diabetes in rural areas in Bangladesh, Pakistan, and Sri Lanka (*n*=2426).

Variables	Total (*n*=2426)	Without diabetes (*n*=1753)	With diabetes (*n*=673)	*P* value
Age (y), mean (SD)	58.8 (11.3)	58.4 (11.6)	59.9 (10.5)	0.003
Men, *n* (%)	864 (35.6)	609 (34.7)	255 (37.9)	0.15
Formal education, *n* (%) (vs. no formal)	1466 (60.4)	959 (54.7)	507 (75.3)	<0.001
Unmarried, *n* (%) (vs. married)	638 (26.3)	468 (26.7)	170 (25.3)	0.47
International wealth index, mean (SD)	58.8 (21.0)	56.1 (21.2)	66.2 (18.4)	<0.001
Smoking, *n* (%)	249 (10.3)	193 (11.0)	56 (8.3)	0.052
Physical activity (MET-min/week), *n* (%)				0.001
<1381	791 (33.0)	558 (32.2)	233 (35.0)	
1381∼5544	810 (33.8)	562 (32.4)	248 (37.3)	
≥5544	798 (33.3)	614 (35.4)	184 (27.7)	
BMI (kg/m^2^), mean (SD)	24.8 (5.0)	24.3 (5.1)	26.0 (4.7)	<0.001
BMI (kg/m^2^), *n* (%)				<0.001
<18.5	214 (8.9)	194 (11.1)	20 (3.0)	
18.5∼23	692 (28.8)	547 (31.3)	145 (22.1)	
23∼27.5	890 (37.0)	617 (35.3)	273 (41.6)	
≥27.5	610 (25.4)	391 (22.4)	219 (33.3)	
Waist circumference^†^ (cm), mean (SD)	88.3 (12.9)	86.5 (12.8)	93.2 (11.9)	<0.001
Waist circumference^†^ (cm), *n* (%)				<0.001
≤Q1	570 (23.5)	506 (28.9)	64 (9.6)	
Q1∼Q2	611 (25.2)	460 (26.3)	151 (22.5)	
Q2∼Q3	584 (24.1)	380 (21.7)	204 (30.5)	
≥Q3	657 (27.1)	406 (23.2)	251 (37.5)	
Systolic blood pressure (mmHg), mean (SD)	145.5 (21.6)	145.1 (21.9)	146.4 (20.9)	0.17
Diastolic blood pressure (mmHg), mean (SD)	88.2 (14.1)	88.5 (14.3)	87.5 (13.6)	0.09
Pulse pressure (mmHg), mean (SD)	57.2 (14.9)	56.6 (14.9)	59.0 (14.6)	<0.001
HDL (mg/dL), mean (SD)	45.2 (13.1)	45.4 (13.3)	44.8 (12.4)	0.38
Triglyceride (mg/dL), median (IQR)	129.4 (94, 183.1)	126.1 (90.7, 177.8)	139.3 (102.1, 202.7)	<0.001
Self-reported CVD, *n* (%)	554 (23.4)	361 (21.1)	193 (29.1)	<0.001
CKD (stage 3 A1 or worse), *n* (%)	899 (38.3)	567 (33.1)	332 (52.5)	<0.001
10-year Framingham CVD risk score 20% or more^‡^, *n* (%)	1052 (44.0)	590 (33.7)	462 (72.1)	<0.001
Country, *n* (%)				<0.001
Bangladesh	872 (35.9)	671 (38.3)	201 (29.9)	
Pakistan	740 (30.5)	588 (33.5)	152 (22.6)	
Sri Lanka	814 (33.6)	494 (28.2)	320 (47.6)	

The number of missing values were 8 for international wealth index, 1 for smoking, 27 for physical activities, 20 for BMI, 4 for waist circumference, 34 for HDL, 35 for triglyceride, 54 for self-reported CVD, 78 for CKD, and 35 for Framingham risk score. SD, standard deviation; Met, metabolic equivalent; BMI, body mass index; HDL, high-density lipoprotein; IQR, interquartile range; CVD, cardiovascular disease; CKD, chronic kidney disease. ^†^Gender-specific quartiles were used: Q1, Q2, and Q3 were 79, 88, and 95 among female and 82, 91, and 98 among male. ^‡^10-year Framingham CVD risk was estimated based on the sex-specific general CVD risk score sheets.

**Table 2:** Overall and country-specific prevalence of diabetes and/or prediabetes among individuals with hypertension in rural communities in Bangladesh, Pakistan, and Sri Lanka (*n*=2426).

	Total (*n*=2426)	Bangladesh (*n*=872)	Pakistan (*n*=740)	Sri Lanka (*n*=814)
Prediabetes or diabetes				
Crude prevalence, *n* (%, 95% CI)	1298 (53.5 (51.5, 55.5))	413 (47.4 (44.0, 50.7))	290 (39.2 (35.6, 42.8))	595 (73.1 (70.0, 76.2))
Age-standardized prevalence, % (95% CI)	—	47.4 (42.7, 52.1)	39.0 (34.4, 43.5)	72.3 (66.0, 78.6)
Age- and gender-standardized prevalence, % (95% CI)	—	47.7 (42.9, 52.5)	38.8 (34.3, 43.4)	72.2 (65.9, 78.6)
Prediabetes only				
Crude prevalence, *n* (%, 95% CI)	625 (25.8 (24.0, 27.5))	212 (24.3 (21.4, 27.2))	138 (18.7 (15.8, 21.5))	275 (33.8 (30.5, 37.1))
Age-standardized prevalence, % (95% CI)	—	24.1 (20.7, 27.4)	18.5 (15.4, 21.6)	34.9 (30.3, 39.4)
Age- and gender-standardized prevalence, % (95% CI)	—	24.2 (20.8, 27.6)	18.7 (15.5, 21.8)	34.4 (29.9, 38.9)
Diabetes only				
Crude prevalence, *n* (%, 95% CI)	673 (27.7 (25.9, 29.5))	201 (23.1 (20.2, 25.9))	152 (20.5 (17.6, 23.5))	320 (39.3 (35.9, 42.7))
Age-standardized prevalence, % (95% CI)	—	23.3 (20.0, 26.6)	20.5 (17.2, 23.8)	37.4 (33.0, 41.8)
Age- and gender-standardized prevalence, % (95% CI)	—	23.5 (20.2, 26.9)	20.2 (16.9, 23.4)	37.8 (33.3, 42.3)

95% CI, 95% confidence interval.

**Table 3:** Factors associated with diabetes among individuals with hypertension in rural communities in Bangladesh, Pakistan, and Sri Lanka.

	Model 1 (*n*=2426)		Model 2 (*n*=2398)		Model 3 (*n*=2398)		Model 4 (*n*=2350)	
	OR (95% CI)	*P* value	OR (95% CI)	*P* value	OR (95% CI)	*P* value	OR (95% CI)	*P* value
Country		<0.001		0.15		0.067		<0.001
Pakistan	1.00		1.00		1.00		1.00	
Bangladesh	1.16 (0.84, 1.61)	0.35	1.12 (0.85, 1.48)	0.41	1.21 (0.91, 1.61)	0.19	1.18 (0.85, 1.62)	0.31
Sri Lanka	2.55 (1.86, 3.49)	<0.001	1.37 (0.99, 1.89)	0.057	1.48 (1.07, 2.06)	0.021	2.32 (1.57, 3.41)	<0.001
Age (y, per 5 y increase)	—	—	1.10 (1.05, 1.16)	<0.001	1.10 (1.04, 1.15)	<0.001	1.05 (0.99, 1.11)	0.090
Women (vs. men)	—	—	0.93 (0.74, 1.16)	0.53	0.93 (0.74, 1.17)	0.53	0.96 (0.74, 1.23)	0.73
Formal education (vs. no formal education)	—	—	1.47 (1.12, 1.92)	0.005	1.35 (1.03, 1.78)	0.028	1.35 (1.02, 1.78)	0.036
Unmarried (vs. married)	—	—	0.87 (0.67, 1.13)	0.28	0.87 (0.67, 1.13)	0.30	0.83 (0.63, 1.08)	0.17
International wealth index (per SD increase)	—	—	1.32 (1.17, 1.50)	<0.001	1.30 (1.15, 1.47)	<0.001	1.30 (1.14, 1.48)	<0.001
BMI (kg/m^2^)	—	—		<0.001		0.55		0.84
<18.5			1.00		1.00		1.00	
18.5∼23			2.29 (1.37, 3.85)	0.002	1.49 (0.86, 2.58)	0.16	1.29 (0.74, 2.26)	0.37
23∼27.5			3.75 (2.25, 6.23)	<0.001	1.47 (0.81, 2.67)	0.20	1.27 (0.69, 2.33)	0.44
≥27.5			4.78 (2.84, 8.07)	<0.001	1.53 (0.81, 2.91)	0.19	1.31 (0.68, 2.52)	0.42
Waist circumference^†^ (cm)	—	—	—	—		<0.001		<0.001
<Q1			—	—	1.00		1.00	
Q1∼Q2			—	—	2.14 (1.47, 3.12)	<0.001	1.97 (1.34, 2.90)	<0.001
Q2∼Q3			—	—	3.45 (2.29, 5.20)	<0.001	3.01 (1.97, 4.59)	<0.001
≥Q3			—	—	3.76 (2.40, 5.89)	<0.001	3.47 (2.18, 5.51)	<0.001
Smoking (vs. no smoking)	—	—	—	—	—	—	0.78 (0.53, 1.13)	0.19
Physical activity (MET-min/week)	—	—	—	—	—	—		0.096
<1381	—	—	—	—	—	—	1.00	
1381–5544	—	—	—	—	—	—	1.03 (0.80, 1.32)	0.82
≥5544	—	—	—	—	—	—	0.79 (0.61, 1.03)	0.083
Pulse pressure (mmHg, per 5 mmHg increase)	—	—	—	—	—	—	1.07 (1.03, 1.11)	<0.001
HDL (mg/dL, per 5 mg/dL increase)	—	—	—	—	—	—	0.90 (0.86, 0.96)	<0.001
Triglyceride (mg/dL, per 5 mg/dL increase)	—	—	—	—	—	—	1.01 (1.01, 1.02)	<0.001

OR, odds ratio; 95% CI, 95% confidence interval; SD, standard deviation; MET, metabolic equivalent task; BMI, body mass index; HDL, high-density lipoprotein. No interaction terms were included in the model. *P* trend for BMI was 0.68 and for waist circumference was <0.001 in model 4. ^†^Gender-specific quartiles were used: Q1, Q2, and Q3 were 79, 88, and 95 among female and 82, 91, and 98 among male.

**Table 4:** Factors associated with diabetes among individuals with hypertension in rural communities in Bangladesh, Pakistan, and Sri Lanka.

	Bangladesh (*n*=866)		Pakistan (*n*=706)		Sri Lanka (*n*=778)	
	OR (95% CI)	*P* Value	OR (95% CI)	*P* Value	OR (95% CI)	*P* Value
Age (y, per 5 y increase)	1.10 (0.99, 1.21)	0.077	0.98 (0.88, 1.11)	0.78	1.04 (0.94, 1.15)	0.43
Women (vs. men)	1.26 (0.76, 2.10)	0.37	0.67 (0.40, 1.12)	0.13	0.86 (0.57, 1.28)	0.45
Formal education (vs. no formal education)	1.15 (0.76, 1.72)	0.51	1.02 (0.61, 1.73)	0.93	3.72 (1.18, 11.77)	0.026
Unmarried (vs. married)	1.19 (0.71, 2.01)	0.50	0.44 (0.24, 0.82)	0.010	0.86 (0.57, 1.29)	0.47
International wealth index (per SD increase)	1.42 (1.10, 1.84)	0.008	1.64 (1.27, 2.12)	<0.001	1.03 (0.83, 1.28)	0.76
Smoking (vs. no smoking)	0.86 (0.44, 1.71)	0.67	1.02 (0.56, 1.87)	0.94	0.65 (0.29, 1.43)	0.28
Physical activity (MET-min/week)		0.81		0.30		0.043
<1381	1.00		1.00		1.00	
1381∼5544	1.10 (0.70, 1.75)	0.68	0.68 (0.39, 1.18)	0.17	1.08 (0.73, 1.60)	0.70
≥5544	0.96 (0.57, 1.63)	0.88	0.74 (0.45, 1.23)	0.24	0.67 (0.44, 1.01)	0.056
BMI (kg/m^2^)		0.82		0.99		0.63
<18.5	1.00		1.00		1.00	
18.5∼23	1.50 (0.39, 5.75)	0.55	1.16 (0.40, 3.34)	0.78	1.49 (0.65, 3.45)	0.34
23∼27.5	1.69 (0.41, 7.05)	0.47	1.08 (0.35, 3.34)	0.89	1.18 (0.47, 2.98)	0.72
≥27.5	1.42 (0.31, 6.59)	0.65	1.07 (0.33, 3.50)	0.91	1.29 (0.48, 3.52)	0.61
Waist circumference^†^ (cm)		0.002		0.019		0.038
<Q1	1.00		1.00		1.00	
Q1∼Q2	2.50 (1.21, 5.14)	0.014	1.81 (0.72, 4.54)	0.21	1.81 (1.01, 3.25)	0.048
Q2∼Q3	4.60 (2.03, 10.42)	<0.001	3.54 (1.38, 9.08)	0.009	2.38 (1.25, 4.52)	0.009
≥Q3	6.01 (2.35, 15.43)	<0.001	3.87 (1.46, 10.26)	0.007	2.72 (1.36, 5.43)	0.005
Pulse pressure (mmHg, per 5 mmHg increase)	1.06 (0.98, 1.14)	0.15	1.16 (1.07, 1.26)	<0.001	1.05 (0.99, 1.11)	0.090
HDL (mg/dL, per 5 mg/dL increase)	1.01 (0.91, 1.12)	0.85	0.91 (0.81, 1.01)	0.088	0.80 (0.73, 0.88)	<0.001
Triglyceride (mg/dL, per 5 mg/dL increase)	1.02 (1.01, 1.03)	0.001	1.01 (1.00, 1.02)	0.011	1.01 (1.00, 1.02)	0.077

OR, odds ratio; 95% CI, 95% confidence interval; SD, standard deviation; MET, metabolic equivalent task; BMI, body mass index; HDL, high-density lipoprotein. Adjusted ORs were reported based on the final model (model 4) in Table 3. *P* trend was 0.99 and <0.001 for BMI and waist circumference in Bangladesh, respectively; *P* trend was 0.95 and 0.005 for BMI and waist circumference in Pakistan, respectively; and *P* trend was 0.99 and 0.007 for BMI and waist circumference in Sri Lanka, respectively. ^†^Gender-specific quartiles were used: Q1, Q2, and Q3 were 79, 88, and 95 among female and 82, 91, and 98 among male.

## Data Availability

The data will be available to the public upon the approval of Trial Steering Committee for COBRA-BPS full-scale study.

## Supplementary Materials

Supplementary Materials Table S1: comparison of baseline characteristics between hypertensive individuals included and excluded from the data analysis. Table S2: baseline characteristics of individuals with hypertension stratified by three countries (*n* = 2426). Table S3: comparison of baseline characteristics between hypertensive individuals with and without diabetes in rural areas in Bangladesh (*n* = 872), Pakistan (*n* = 740), and Sri Lanka (*n* = 814). Table S4: ratio of odds ratios (RORs) between countries for variables that had significant interactions with country. Table S5: management of diabetes among hypertensive individuals with diabetes (*n* = 673). Table S6: use of glucose-lowering medications by hypertensive individuals with diabetes in rural communities in Bangladesh, Pakistan, and Sri Lanka (*n* = 673). Figure S1: study flow chart of hypertensive individuals included in the study on prediabetes and diabetes.

## References

[1] Shaw J. E., Sicree R. A., Zimmet P. Z. (2010). Global estimates of the prevalence of diabetes for 2010 and 2030. *Diabetes Research and Clinical Practice*.

[2] Long A. N., Dagogo-Jack S. (2011). Comorbidities of diabetes and hypertension: mechanisms and approach to target organ protection. *The Journal of Clinical Hypertension*.

[3] Federation ID (2017). *IDF Diabetes Atlas*.

[4] Tandon N., Anjana R. M., Mohan V. (2018). The increasing burden of diabetes and variations among the states of India: the Global Burden of Disease Study 1990–2016. *The Lancet Global health*.

[5] Islam A., Zaffar Tahir M. (2002). Health sector reform in South Asia: new challenges and constraints. *Health Policy*.

[6] Gupta L. S., Wu C. C., Young S., Perlman S. E. (2011). Prevalence of diabetes in New York city, 2002–2008: figure 1. *Diabetes Care*.

[7] Agyemang C., Kunst A. E., Bhopal R. (2011). Diabetes prevalence in populations of South asian Indian and african origins. *Epidemiology*.

[8] Misra A., Tandon N., Ebrahim S. (2017). Diabetes, cardiovascular disease, and chronic kidney disease in South Asia: current status and future directions. *BMJ*.

[9] Banerjee A. T., Shah B. R. (2018). Differences in prevalence of diabetes among immigrants to Canada from South Asian countries. *Diabetic Medicine*.

[10] Admiraal W. M., Holleman F., Snijder M. B. (2014). Ethnic disparities in the association of impaired fasting glucose with the 10-year cumulative incidence of type 2 diabetes. *Diabetes Research and Clinical Practice*.

[11] Hughes A. D., Bathula R., Park C. (2013). Microcirculatory rarefaction in South Asians—a potential mechanism for increased cardiovascular risk and diabetes. *PLoS One*.

[12] Gunarathne A., Patel J. V., Kausar S., Gammon B., Hughes E. A., Lip G. Y. H. (2009). Glycemic status underlies increased arterial stiffness and impaired endothelial function in migrant South Asian stroke survivors compared to european Caucasians. *Stroke*.

[13] Bhargava S. K., Sachdev H. S., Fall C. H. D. (2004). Relation of serial changes in childhood body-mass index to impaired glucose tolerance in young adulthood. *New England Journal of Medicine*.

[14] Ebrahim S., Kinra S., Bowen L. (2010). The effect of rural-to-urban migration on obesity and diabetes in India: a cross-sectional study. *PLoS Medicine*.

[15] Goryakin Y., Lobstein T., James W. P. T., Suhrcke M. (2015). The impact of economic, political and social globalization on overweight and obesity in the 56 low and middle income countries. *Social Science & Medicine*.

[16] Viswanathan V., Madhavan S., Rajasekar S., Chamukuttan S., Ambady R. (2006). Urban-rural differences in the prevalence of foot complications in South-Indian diabetic patients. *Diabetes Care*.

[17] Palmieri V., Bella J. N., Arnett D. K. (2001). Effect of type 2 diabetes mellitus on left ventricular geometry and systolic function in hypertensive subjects. *Circulation*.

[18] Jafar T. H., Hatcher J., Poulter N. (2009). Community-based interventions to promote blood pressure control in a developing country. *Annals of Internal Medicine*.

[19] Qin X., Li J., Zhang Y. (2012). Prevalence and associated factors of diabetes and impaired fasting glucose in Chinese hypertensive adults aged 45 to 75 years. *PLoS One*.

[20] Yu S., Sun Z., Zheng L., Guo X., Yang H., Sun Y. (2015). Prevalence of diabetes and impaired fasting glucose in hypertensive adults in rural China: far from leveling-off. *International Journal of Environmental Research and Public Health*.

[21] Alves-Cabratosa L., García-Gil M., Comas-Cufí M. (2016). Diabetes and new-onset atrial fibrillation in a hypertensive population. *Annals of Medicine*.

[22] Safar M. E., Gnakamene J. B., Bahous S. A., Yannoutsos A., Thomas F. (2017). Longitudinal study of hypertensive subjects with type 2 diabetes mellitus: overall and cardiovascular risk. *Hypertension*.

[23] Huang X. B., Tang W. W., Liu Y. (2017). Prevalence of diabetes and unrecognized diabetes in hypertensive patients aged 40 to 79 years in southwest China. *PLoS One*.

[24] Aksnes T. A., Kjeldsen S. E., Rostrup M., Störset Ö., Hua T. A., Julius S. (2008). Predictors of new-onset diabetes mellitus in hypertensive patients: the VALUE trial. *Journal of Human Hypertension*.

[25] Yasuno S., Ueshima K., Oba K. (2010). Is pulse pressure a predictor of new-onset diabetes in high-risk hypertensive patients?: a subanalysis of the candesartan antihypertensive survival evaluation in Japan (CASE-J) trial. *Diabetes Care*.

[26] Gupta A. K., Dahlof B., Dobson J. (2008). Determinants of new-onset diabetes among 19,257 hypertensive patients randomized in the anglo-scandinavian cardiac outcomes trial-blood pressure lowering arm and the relative influence of antihypertensive medication. *Diabetes Care*.

[27] Jayawardena R., Ranasinghe P., Byrne N. M., Soares M. J., Katulanda P., Hills A. P. (2012). Prevalence and trends of the diabetes epidemic in South Asia: a systematic review and meta-analysis. *BMC Public Health*.

[28] Chan J. C. N., Malik V., Jia W. (2009). Diabetes in Asia. *JAMA*.

[29] Jafar T. H., Jehan I., de Silva H. A. (2017). Multicomponent intervention versus usual care for management of hypertension in rural Bangladesh, Pakistan and Sri Lanka: study protocol for a cluster randomized controlled trial. *Trials*.

[30] Smits J., Steendijk R. (2015). The international wealth index (IWI). *Social Indicators Research*.

[31] Craig C. L., Marshall A. L., Sjöström M. (2003). International physical activity questionnaire: 12-country reliability and validity. *Medicine & Science in Sports & Exercise*.

[32] D’Agostino R. B., Vasan R. S., Pencina M. J. (2008). General cardiovascular risk profile for use in primary care: the Framingham Heart Study. *Circulation*.

[33] Levey A. S., Stevens L. A., Schmid C. H. (2009). A new equation to estimate glomerular filtration rate. *Annals of Internal Medicine*.

[34] IDF (2006). *The IDF Consensus Worldwide Definition of the Metabolic Syndrome*.

[35] WHO Expert Consultation (2004). Appropriate body-mass index for Asian populations and its implications for policy and intervention strategies. *The Lancet*.

[36] Cornelis M. C., Chiuve S. E., Glymour M. M. (2014). Bachelors, divorcees, and widowers: does marriage protect men from type 2 diabetes?. *PLoS One*.

[37] Jaccard J. (2001). *Interaction Effects in Logistic Regression*.

[38] Katulanda P., Constantine G. R., Mahesh J. G. (2008). Prevalence and projections of diabetes and pre-diabetes in adults in Sri Lanka-Sri Lanka Diabetes, Cardiovascular Study (SLDCS). *Diabetic Medicine*.

[39] Akter S., Rahman M. M., Abe S. K., Sultana P. (2014). Prevalence of diabetes and prediabetes and their risk factors among Bangladeshi adults: a nationwide survey. *Bulletin of the World Health Organization*.

[40] Jafar T. H., Levey A. S., White F. M. (2004). Ethnic differences and determinants of diabetes and central obesity among South Asians of Pakistan. *Diabetic Medicine*.

[41] Creatore M. I., Moineddin R., Booth G. (2010). Age- and sex-related prevalence of diabetes mellitus among immigrants to Ontario, Canada. *Canadian Medical Association Journal*.

[42] Deepa M., Grace M., Binukumar B. (2015). High burden of prediabetes and diabetes in three large cities in South Asia: the center for cArdio-metabolic risk reduction in South Asia (CARRS) study. *Diabetes Research and Clinical Practice*.

[43] Jayasuriya D., Jayasuriya R., Tay A. K., Silove D. (2016). Associations of mental distress with residency in conflict zones, ethnic minority status, and potentially modifiable social factors following conflict in Sri Lanka: a nationwide cross-sectional study. *The Lancet Psychiatry*.

[44] Hackett R. A., Steptoe A. (2017). Type 2 diabetes mellitus and psychological stress—a modifiable risk factor. *Nature Reviews Endocrinology*.

[45] Heraclides A., Chandola T., Witte D. R., Brunner E. J. (2009). Psychosocial stress at work doubles the risk of type 2 diabetes in middle-aged women: evidence from the Whitehall II study. *Diabetes Care*.

[46] Triches C. B., Mayer S., Quinto B. M. R., Batista M. C., Zanella M. T. (2018). Association of endothelial dysfunction with cardiovascular risk factors and new-onset diabetes mellitus in patients with hypertension. *Journal of Clinical Hypertension*.

[47] Daniels S. I., Chambers J. C., Sanchez S. S. (2018). Elevated levels of organochlorine pesticides in South Asian immigrants are associated with an increased risk of diabetes. *Journal of the Endocrine Society*.

[48] Qiao Q., Nyamdorj R. (2010). Is the association of type II diabetes with waist circumference or waist-to-hip ratio stronger than that with body mass index?. *European Journal of Clinical Nutrition*.

[49] Dale C. E., Fatemifar G., Palmer T. M. (2017). Causal associations of adiposity and body fat distribution with coronary heart disease, stroke subtypes, and type 2 diabetes mellitus. *Circulation*.

[50] Ruderman N., Chisholm D., Pi-Sunyer X., Schneider S. (1998). The metabolically obese, normal-weight individual revisited. *Diabetes*.

[51] Misra A., Vikram N. K. (2003). Clinical and pathophysiological consequences of abdominal adiposity and abdominal adipose tissue depots. *Nutrition*.

[52] Goossens G. H., Vogel M. A. A., Vink R. G., Mariman E. C., van Baak M. A., Blaak E. E. (2018). Adipose tissue oxygenation is associated with insulin sensitivity independently of adiposity in obese men and women. *Diabetes, Obesity and Metabolism*.

[53] Chandalia M., Abate N., Garg A., Stray-Gundersen J., Grundy S. M. (1999). Relationship between generalized and upper body obesity to insulin resistance in Asian Indian men. *Journal of Clinical Endocrinology & Metabolism*.

[54] Lear S. A., Humphries K. H., Kohli S., Chockalingam A., Frohlich J. J., Birmingham C. L. (2007). Visceral adipose tissue accumulation differs according to ethnic background: results of the Multicultural Community Health Assessment Trial (M-CHAT). *American Journal of Clinical Nutrition*.

[55] DeBoer M. D., Filipp S. L., Gurka M. J. (2018). Use of a metabolic syndrome severity Z score to track risk during treatment of prediabetes: an analysis of the diabetes prevention program. *Diabetes Care*.

[56] Williams J., Allen L., Wickramasinghe K., Mikkelsen B., Roberts N., Townsend N. (2018). A systematic review of associations between non-communicable diseases and socioeconomic status within low- and lower-middle-income countries. *Journal of Global Health*.

[57] GDP, PPP (Current International $) (October 2018). *International Comparison Program Database*.

[58] Tuomilehto J., Rastenyte D., Birkenhäger W. H. (1999). Effects of calcium-channel blockade in older patients with diabetes and systolic hypertension. *New England Journal of Medicine*.

[59] Ali M. K., Bullard K. M., Saaddine J. B., Cowie C. C., Imperatore G., Gregg E. W. (2013). Achievement of goals in U.S. Diabetes care, 1999–2010. *New England Journal of Medicine*.

[60] (October 2018). Algorithm for blood glucose lowering therapy in adults with type 2 diabetes, 2017. https://www.nice.org.uk/guidance/ng28/resources/algorithm-for-blood-glucose-lowering-therapy-in-adults-with-type-2-diabetes-pdf-2185604173.

[61] Chamberlain J. J., Rhinehart A. S., Shaefer C. F., Neuman A. (2016). Diagnosis and management of diabetes: synopsis of the 2016 American diabetes association standards of medical care in diabetes. *Annals of Internal Medicine*.

[62] National Institute for Health and Care Excellence (2014). *Lipid Modification: Cardiovascular Risk Assessment and the Modification of Blood Lipids for the Primary and Secondary Prevention of Cardiovascular Disease, Clinical Guideline CG181*.

[63] Chowdhury M. A., Uddin M. J., Khan H. M., Haque M. R. (2015). Type 2 diabetes and its correlates among adults in Bangladesh: a population based study. *BMC Public Health*.

[64] Wijewardene K., Mohideen M. R., Mendis S. (2005). Prevalence of hypertension, diabetes and obesity: baseline findings of a population based survey in four provinces in Sri Lanka. *Ceylon Medical Journal*.

