# Supplementary material for: Regional Variation in Comorbid Prediabetes and Diabetes and Associated Factors among Hypertensive Individuals in Rural Bangladesh, Pakistan, and Sri Lanka
Source: J Obes. 2019 Apr 30;2019:4914158. doi: 10.1155/2019/4914158 (PMC6515018; doi:10.1155/2019/4914158)

Supplementary Material

Table S1: Comparison of baseline characteristics between hypertensive individuals included and excluded from the data analysis

Variables	Total N (n=2643)	Sample included (n=2426)	Sample excluded (n=217)	P value
Country, n (%)				<0.001
Bangladesh		872 (35.9)	23 (10.6)	
Pakistan		740 (30.5)	154 (71.0)	
Sri Lanka		814 (33.6)	40 (18.4)	
Age, (y, mean (SD))		58.8 (11.3)	58.2 (13.0)	0.52
Men, n (%)		864 (35.6)	79 (36.4)	0.82
Education, n (%)				<0.001
Formal education		1466 (60.4)	91 (41.9)	
No Formal education		960 (39.6)	126 (58.1)	
Marrital status, n (%)				<0.001
Unmarried		638 (26.3)	80 (36.9)	
Married		1788 (73.7)	137 (63.1)	
International Wealth Index, mean (SD)	2634	58.9 (21.0)	53.3 (21.3)	<0.001
Smoking, n (%)		249 (10.3)	25 (11.5)	0.56
Physical activities (MET-min/week), n (%)	2611			0.013
<1381		791 (33.0)	91 (42.9)	
1381~5544		810 (33.8)	63 (29.7)	
≥5544		798 (33.3)	58 (27.4)	
BMI, mean (SD)	2588	24.8 (5.0)	24.4 (5.7)	0.39
BMI (kg/m2) , n(%)	2588			0.046
<18.5		214 (8.9)	25 (13.7)	
18.5~23.0		692 (28.8)	60 (33.0)	
23.0~27.5		890 (37.0)	54 (29.7)	
≥27.5		610 (25.4)	43 (23.6)	
Waist circumference†(cm), mean(SD)	2633	88.3 (12.9)	86.5 (14.2)	0.06
Waist circumference† (cm), n (%)	2633			0.01
<Q1		570 (23.5)	67 (31.8)	
Q1~Q2		611 (25.2)	52 (24.6)	
Q2~Q3		584 (24.1)	33 (15.6)	
≥Q3		657 (27.1)	59 (28.0)	
Pulse pressure (mmHg, mean (SD))		57.2 (14.9)	57.3 (15.7)	0.98

Abbreviation: SD, standard deviation; Met, metabolic equivalent; BMI, body mass index. Comparison for high density lipoprotein (HDL) and triglyceride was not reported because only 5 individuals with data on HDL and triglyceride among the excluded sample.

† Gender-specific quartiles were used: Q1 , Q2 and Q3 were 79,88 ,and 95 among female; and 82,91, and 98 among male.

Table S2. Baseline characteristics of individuals with hypertension stratified by three countries (n=2426)

Variables	Bangladesh (n=872)	Pakistan (n=740)	Sri Lanka (n=814)	P value
Age (y), mean (SD)	56.7 (11.2)	56.8 (11.3)	62.9 (10.4)	<0.001
Men, n (%)	316 (36.2)	289 (39.1)	259 (31.8)	0.011
Education, n (%)				<0.001
Formal education	454 (52.1)	227 (30.7)	785 (96.4)	
No Formal education	418 (47.9)	513 (69.3)	29 (3.6)	
Marrital status, n (%)				<0.001
Unmarried	182 (20.9)	184 (24.9)	272 (33.4)	
Married	690 (79.1)	556 (75.1)	542 (66.6)	
International Wealth Index, mean (SD)	50.7 (17.0)	52.4 (20.9)	73.6 (16.9)	<0.001
Smoking, n (%)	105 (12.1)	101 (13.7)	43 (5.3)	<0.001
Physical activities (MET-min/week),				<0.001
<1381	213 (24.4)	320 (44.0)	258 (32.3)	
1381~5544	386 (44.3)	158 (21.7)	266 (33.3)	
≥5544	273 (31.3)	249 (34.3)	276 (34.5)	
BMI (kg/m2), mean (SD)	24.2 (4.0)	25.1 (6.2)	25.1 (4.7)	<0.001
BMI (kg/m2), n(%)				<0.001
<18.5	66 (7.6)	94 (12.7)	54 (6.8)	
18.5~23.0	276 (31.8)	201 (27.2)	215 (26.9)	
23.0~27.5	359 (41.3)	225 (30.5)	306 (38.3)	
≥27.5	168 (19.3)	218 (29.5)	224 (28.0)	
Waist circumference† (cm), mean (SD)	86.2 (10.8)	89.3 (15.3)	89.8 (12.3)	0.001
Waist circumference† (cm), n(%)				<0.001
<Q1	224 (25.8)	187 (25.3)	159 (19.5)	
Q1~Q2	264 (30.3)	162 (22.0)	185 (22.7)	
Q2~Q3	233 (26.8)	145 (19.7)	206 (25.4)	
≥Q3	149 (17.1)	244 (33.1)	264 (32.3)	
SBP (mmHg), mean (SD)	141.6 (20.7)	148.7 (21.0)	146.7 (22.5)	<0.001
DBP (mmHg), mean (SD)	87.1 (13.7)	91.3 (14.0)	86.7 (14.4)	<0.001
Pulse pressure (mmHg), mean (SD)	54.5 (13.7)	57.5 (15.4)	60.0 (15.0)	<0.001
HDL (mg/dL) , mean (SD)	38.2 (10.4)	42.6 (12.7)	55.3 (9.3)	<0.001
Triglyceride (mg/dL), median (IQR)	145.9 (105.4,207.9)	137 (98.0,197.0)	108.7 (85.1,143.4)	<0.001
Self-reported CVD, n (%)	282 (34.0)	93 (12.6)	179 (22.2)	<0.001
CKD (Stage 3 A1 or worse), n(%)	316 (36.4)	117 (17.1)	446 (58.5)	<0.001
Framingham CVD risk score 20% or more, n (%)	380 (43.8)	280 (38.5)	392 (49.2)	<0.001

Abbreviation: SD, standard deviation; Met, metabolic equivalent; BMI, body mass index; SBP, systolic blood pressure; DBP, diastolic blood pressure; HDL, high density lipoprotein, IQR, inter quartile range; CVD, cardiovascular disease; CKD, chronic kidney disease.The number of missing values were 8 for international wealth index, 1 for smoking, 27 for physical activities, 20 for BMI, 4 for waist circumference, 34 for HDL, 35 for triglyceride, 54 for self-reported CVD, 78 for CKD, and 35 for Framingham risk score.

† Gender-specific quartiles were used: Q1 , Q2 and Q3 were 79,88 ,and 95 among female; and 82,91, and 98 among male.

Table S3 Comparison of baseline characteristics between hypertensive individuals with and without diabetes in rural areas in Bangladesh (n=872), Pakistan (n=740), and Sri Lanka (n=814)

Variables	Bangladesh			Pakistan			Sri Lanka		
	Without diabetes (n=671)	With diabetes (n=201)	P value	Without diabetes (n=588)	With diabetes (n=152)	P value	Without diabetes (n=494)	With diabetes (n=320)	P value
Age (y), mean (SD)	56.5 (11.3)	57.4 (11.0)	0.36	56.8 (11.4)	56.9 (10.7)	0.95	62.9 (11.1)	62.9 (9.3)	0.97
Men, n (%)	247 (36.8)	69 (34.3)	0.52	213 (36.2)	76 (50.0)	0.002	149 (30.2)	110 (34.4)	0.21
Education, n (%)			0.014			<0.001			0.004
Formal education	334 (49.8)	120 (59.7)		156 (26.5)	71 (46.7)		469 (94.9)	316 (98.7)	
No Formal education	337 (50.2)	81 (40.3)		432 (73.5)	81 (53.3)		25 (5.1)	4 (1.3)	
Marrital status, n (%)			0.69			0.004			0.45
Unmarried	138 (20.6)	44 (21.9)		160 (27.2)	24 (15.8)		170 (34.4)	102 (31.9)	
Married	533 (79.4)	157 (78.1)		428 (72.8)	128 (84.2)		324 (65.6)	218 (68.1)	
International Wealth Index, mean (SD)	49.1 (17.2)	55.8 (15.4)	<0.001	50.3 (21.0)	60.7 (18.1)	<0.001	72.4 (17.5)	75.3 (15.7)	0.014
Smoking, n(%)	87 (13.0)	18 (9.0)	0.13	78 (13.3)	23 (15.1)	0.55	28 (5.7)	15 (4.7)	0.54
Physical activities (MET-min/week), n(%)			0.20			0.18			0.013
<1381	163 (24.3)	50 (24.9)		246 (42.6)	74 (49.3)		149 (30.7)	109 (34.7)	
1381~5544	288 (42.9)	98 (48.8)		124 (21.5)	34 (22.7)		150 (30.9)	116 (36.9)	
≥5544	220 (32.8)	53 (26.4)		207 (35.9)	42 (28.0)		187 (38.5)	89 (28.3)	
BMI (kg/m2), mean (SD)	23.7 (4.1)	25.7 (3.5)	<0.001	24.6 (6.1)	27.1 (5.9)	<0.001	24.8 (4.8)	25.7 (4.6)	0.005
BMI (kg/m2), n(%)			<0.001			<0.001			0.005
<18.5	63 (9.4)	3 (1.5)		87 (14.8)	7 (54.6)		44 (9.0)	10 (3.3)	
18.5~23.0	236 (35.2)	40 (20.2)		173 (29.5)	28 (18.5)		138 (28.1)	77 (25.0)	
23.0~27.5	262 (39.1)	97 (49.0)		171 (29.1)	54 (35.8)		184 (37.5)	122 (39.6)	
≥27.5	110 (16.4)	58 (29.3)		156 (26.6)	62 (41.1)		125 (25.5)	99 (32.1)	
Waist circumference† (cm), mean (SD)	84.7 (10.8)	91.2 (89.9)	<0.001	87.3 (14.8)	97.0 (15.1)	<0.001	88.0 (12.5)	92.6 (11.4)	<0.001
Waist circumference† (cm), n(%)			<0.001			<0.001			<0.001

<Q1	208 (31.0)	16 (8.0)		174 (29.6)	13 (8.6)		124 (25.1)	35 (10.9)	
Q1~Q2	211 (31.5)	53 (26.6)		137 (23.3)	25 (16.6)		112 (22.7)	73 (22.8)	
Q2~Q3	158 (23.6)	75 (37.7)		105 (17.9)	40 (26.5)		117 (23.7)	89 (27.8)	
≥Q3	94 (14.0)	55 (27.6)		171 (29.1)	73 (48.3)		141 (28.5)	123 (38.4)	
SBP (mmHg), mean (SD)	141.6 (21.1)	141.4 (19.3)	0.92	148.2 (21.3)	151.0 (19.6)	0.14	146.2 (23.0)	147.4 (21.8)	0.45
DBP (mmHg), mean (SD)	87.3 (14.1)	86.4 (12.2)	0.38	91.5 (14.2)	90.6 (13.1)	0.50	86.8 (14.4)	86.7 (14.4)	0.94
Pulse pressure (mmHg), mean (SD)	54.3 (13.7)	55.0 (14.0)	0.51	56.7 (15.7)	60.5 (13.9)	0.009	59.5 (15.1)	60.7 (15.0)	0.23
HDL (mg/dL) , mean (SD)	38.6 (10.4)	36.8 (10.5)	0.031	43.4 (13.2)	39.2 (10.1)	<0.001	56.8 (8.9)	52.7 (9.5)	<0.001
Triglyceride (mg/dL), median (IQR)	140.7 (99.8,195.4)	166.7 (123.6,247.2)	<0.001	132.0 (94.0,186.0)	165.5 (118.0,262.0)	<0.001	104.9 (83.0,137.7)	115.7 (90.4,158.8)	<0.001
Self-reported CVD, n (%)	203 (31.9)	79 (40.9)	0.021	60 (10.3)	33 (21.7)	<0.001	98 (20.1)	81 (25.4)	0.076
CKD (Stage 3 A1 or worse), n (%)	218 (32.5)	98 (49.8)	<0.001	81 (14.7)	36 (27.1)	<0.001	268 (54.3)	198 (65.4)	0.002
Framingham CVD risk score 20% or more, n (%)	250 (37.3)	130 (66.3)	<0.001	175 (29.9)	105 (73.9)	<0.001	165 (33.4)	227 (74.9)	<0.001

Abbreviation: SD, standard deviation; Met, metabolic equivalent; BMI, body mass index; SBP, systolic blood pressure; DBP, diastolic blood pressure; HDL, high density lipoprotein, IQR, inter quartile range; CVD, cardiovascular disease; CKD, chronic kidney disease

Number of missing data: Bangladesh: one missing value for smoking, 3 missing BMI, 2 missing waist circumference, 4 missing HDL, 5 missing triglyceride , 43 missing for self-reported CVD, 4 missing CKD status, 5 missing Framingham CVD risk score. Pakistan: 7 missing for international wealth index, 13 for physical activities, 2 for BMI, 2 for waist circumference, 13 for HDL and triglyceride, 4 for self-reported CVD,57 for CKD, and 13 for Framingham CVD risks score. Sri Lanka: 14 missing for physical activities, 15 for BMI, 17 for HDL and triglyceride, 1 for international wealth index, and 7 for self-reported CVD, 17 for CKD, and 17 for Framingham CVD risks score.

† Gender-specific quartiles were used: Q1 , Q2 and Q3 were 79,88 ,and 95 among female; and 82,91, and 98 among male.

Table S4 Ratio of odds ratios (RORs) between countries for variables that had significant interactions with country.

Variables	P value for interaction †	Bangladesh vs. Pakistan	Pakistan vs. Sri Lanka	Bangladesh vs. Sri Lanka
		ROR (95% CI)(P value)	ROR (95% CI) (P value)	ROR (95% CI) (P value)
Unmarried (VS married)	0.011	2.92 (1.45, 5.87) (0.003)	0.55 (0.29, 1.05) (0.069)	1.60 (0.92, 2.78) (0.10)
IWI (per SD increase)	0.038	0.93 (0.67, 1.28) (0.64)	1.47 (1.07, 2.01) (0.017)	1.36 (0.99, 1.86) (0.055)
HDL (mg/dL ,per 5 mg/dL increase)	0.002	1.11 (0.97, 1.27) (0.12)	1.13 (0.99, 1.29) (0.075)	1.25 (1.11, 1.42) (<0.001)

Abbreviations: 95%CI, 95% confidence interval; IWI, International Wealth Index; SD, standard deviation, HDL, high density lipoprotein

† P value for interaction with country in model 4 based on overall sample in table 3

Table S5. Management of diabetes among hypertensive individuals with diabetes (n=673)

	Overall (n=673)	Bangladesh (n=201)	Pakistan (n=152)	Sri Lanka (n=320)
Awareness of diabetes †, n (% (95% CI))	500 (74.3 (70.9, 77.7))	164 (81.6 (76.0, 87.2))	80 (52.6 (44.4, 60.9))	256 (80.0 (75.5, 84.5))
Controlled blood pressure ǂ , n (% (95% CI))	221 (32.8 (29.2, 36.5))	84 (41.8 (34.7, 48.8))	26 (17.1 (10.7, 23.4))	111 (34.7 (29.3, 40.1))
Statin use , n (% (95% CI))	160 (23.8 (20.5, 27.1))	14 (7.0 (3.2, 10.7))	13 (8.6 (3.8, 13.3))	133 (41.6 (36.0, 47.1))

ǂSelf-reported physician diagnosis of diabetes

ǂControlled blood pressure: SBP<140mmHg or DBP<90 mmHg

Table S6. Use of glucose lowering medications by hypertensive individuals with diabetes in rural communities in Bangladesh, Pakistan, and Sri Lanka (n=673)

	Overall (n=673)	Bangladesh (n=201)	Pakistan (n=152)	Sri Lanka (n=320)
Any antidiabetics use, n (% (95% CI))	462 (68.7(65.1,72.2))	148 (73.6 (67.3,80.0))	70 (46.1 (37.8,54.3))	244 (76.3 (71.4,81.1))
Biguanide use, n (% (95% CI))	328 (48.7 (44.9,52.6))	71 (35.3 (28.5,42.2))	49 (32.2 (24.5,40.0))	208 (65.0 (59.6,70.4))
Sulfonylurea use, n (% (95% CI))	237 (35.2 (31.5,38.9))	73 (36.3 (29.4,43.2))	43 (28.3 (20.8,35.8))	121 (37.8 (32.3,43.3))
Other antidiabetics use, n (% (95% CI))	89 (13.2 (10.6,15.9))	51 (25.4 (19.1,31.6))	6 (4.0 (0.5,7.4))	32 (10.0 (6.6,13.4))
Number of antidiabetics use,n (% (95% CI))				
0	211(31.5 (27.8,34.9))	53 (26.4 (20.0,32.7))	82 (54.0 (45.7,62.2))	76 (23.8 (18.9,28.6))
1	270 (40.1 (36.3,43.9))	91 (45.3 (38.1,52.4))	40 (26.3 (19.0,33.7))	139 (43.4 (37.9,49.0)
2 or more	192 (28.5 (25.0,32.0))	57 (28.4 (21.9,34.8))	30 (19.7 (13.1,26.4))	105 (32.8 (27.5,38.1))

Abbreviations: 95%CI, 95% confidence interval

Supplementary Figure

Figure S1: Study flow chart of hypertensive individuals included in the study on **prediabetes and diabetes**

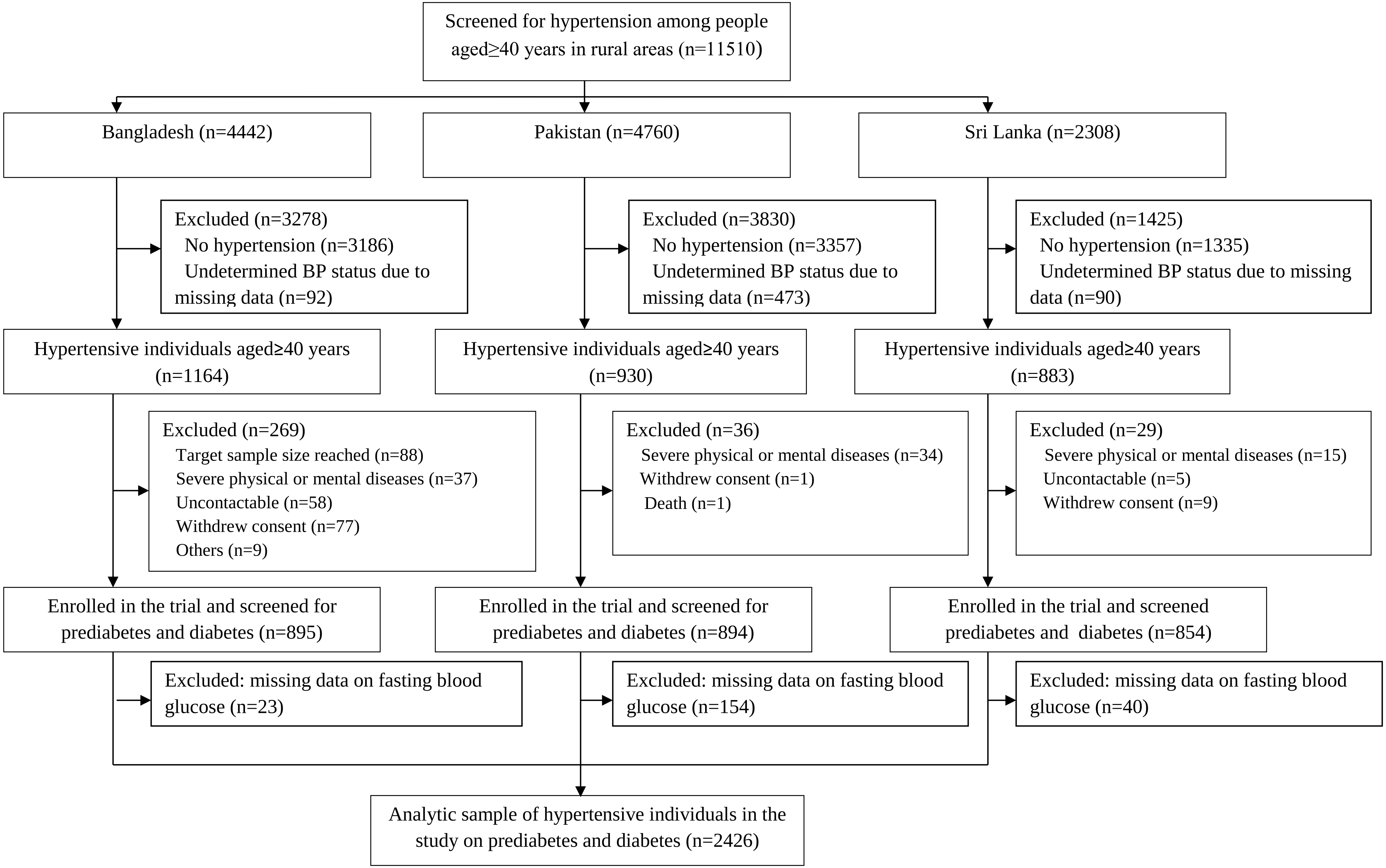

Supplement: Supplementary Materials — Table S1: comparison of baseline characteristics between hypertensive individuals included and excluded from the data analysis. Table S2: baseline characteristics of individuals with hypertension stratified by three countries (n = 2426). Table S3: comparison of baseline characteristics between hypertensive individuals with and without diabetes in rural areas in Bangladesh (n = 872), Pakistan (n = 740), and Sri Lanka (n = 814). Table S4: ratio of odds ratios (RORs) between countries for variables that had significant interactions with country. Table S5: management of diabetes among hypertensive individuals with diabetes (n = 673). Table S6: use of glucose-lowering medications by hypertensive individuals with diabetes in rural communities in Bangladesh, Pakistan, and Sri Lanka (n = 673). Figure S1: study flow chart of hypertensive individuals included in the study on prediabetes and diabetes. [file 4914158.f1.pdf]
